# Minimizing policy-biased appraisals of the evidence on cannabis and psychosis

**DOI:** 10.3389/fpsyt.2022.1047860

**Published:** 2023-01-09

**Authors:** Wayne Hall

**Affiliations:** The National Centre for Youth Substance Use Research, The University of Queensland, Brisbane, QLD, Australia

**Keywords:** cannabis, psychosis, causal inference, policy-biased appraisals, policy implications

## Abstract

Appraisals of the evidence on the relationship between cannabis use and psychosis are often biased by appraisers' pre-existing views on whether adult cannabis use should or should not be legal. This viewpoint gives examples of such policy-biased appraisals and suggests strategies for avoiding them.

The debate about whether cannabis use is a contributory cause of psychosis is often seen as critical to the policy debate about whether adults should be legally able to use cannabis (1). Proponents on either side of the debate implicitly assume that the case for cannabis legalization is weakened if we accept that the relationship is causal. Supporters of retaining criminal penalties for adult cannabis use often support their case by arguing that cannabis is a cause of psychosis [e.g., (2, 3)] while some who support more liberal cannabis policies argue that the association is not causal [e.g., (4)]. This alignment of views can lead to policy-biased appraisals of evidence, i.e., appraisals in which evidence is selectively interpreted to support a pre-existing policy commitment. We need to disentangle our appraisals of the empirical evidence from our policy commitments.

## Defining some key terms

A psychosis is a serious mental disorder in which a person, most often a young adult, experiences hallucinations (e.g., accusatory voices) and develops delusional beliefs that other people want to harm them. Persons with these symptoms may have impaired cognitive and social functioning that interferes with their ability to form close personal relationships, prevents them from completing their education, and makes it difficult for them to earn a living (5).

In this article, regular cannabis use refers to the daily or near daily use of cannabis. This pattern of cannabis use predicts an increased risk of psychosis, especially when it begins in adolescence and continues into adult life.

The hypothesis that cannabis is a cause of psychosis does not imply that cannabis use is a necessary or a sufficient condition for developing a psychosis. It is not necessary because many persons who develop psychoses have not used cannabis; it not sufficient because only a minority of cannabis users develop a psychosis (6).

A more plausible hypothesis is that regular cannabis use is a *contributory cause* of psychosis (1). On this hypothesis, regular cannabis use is one of a combination of factors that increase the risk of psychosis, or brings forward the onset of the illness in persons who are at increased risk of developing a psychosis, e.g., by having a parent or sibling with a psychosis. The factors with which regular cannabis use may interact include genetic vulnerabilities to develop a psychosis and environmental exposures that increase the risk of psychosis, such as childhood abuse and other unknown factors (1).

## The case for a contributory causal relationship

In longitudinal studies of representative samples of young people, there is a consistent evidence that daily or near daily cannabis use in adolescence and young adulthood predicts an increased risk of psychotic symptoms or a diagnosis of a schizophreniform disorder (7–10).

Those who argue that cannabis use is a contributory cause of psychosis use [e.g., (1, 9–11)]. point to coherence of a set of interlocking kinds of evidence, namely, that cannabis use typically precedes the onset of psychosis, and the earlier cannabis use begins, the heavier cannabis use is, and the longer regular use lasts, the greater the risk of experiencing psychotic symptoms or developing a psychotic disorder (9–11). The principal psychoactive ingredient in cannabis—tetrahydrocannabinol (THC)—acts upon CB_1_ cannabinoid receptors in the brain (8, 12) and the cannabinoid system that they comprise, in turn, interacts with dopaminergic and other neurotransmitters systems that have been implicated in the production of psychotic symptoms (12). When THC is given under double blind conditions, it also produces dose related increases in psychotic symptoms in persons who do and do not have a psychosis (13, 14). Cannabis users who develop schizophrenia have a worse clinical course, if they continue to use cannabis than do peers with a psychotic illness who cease using cannabis (15, 16).

### Alternatives to a causal explanation

Those who are skeptical that cannabis is a contributory cause of psychosis suggest two alternative explanations of the association.

The first is that psychotic symptoms are a cause of early and heavy cannabis use rather than vice-versa (17). A popular common version of this hypothesis is that persons with early symptoms of psychosis use cannabis to medicate its symptoms, such as depression, or the side effects of the medications used to treat psychosis (4). This hypothesis would explain why regular cannabis use is common among newly incident cases of psychosis (9).

The second possibility is that the association reflects the effects of shared risk factors for early and regular cannabis use and for psychosis. According to this hypothesis, shared risk factors increase (1) the risk of early and regular cannabis use in young adulthood and (2) increase the risk of developing a psychosis. These shared risk factors could be environmental factors such as childhood abuse, genetic factors, or some combination of the two (7).

### The self-medication hypothesis

The support for the self-medication hypothesis is weaker than that shared risk factors hypothesis. First, people with psychoses who use cannabis provide the same reasons for using cannabis as persons who do not have a psychosis, namely, its effects feel good, they want to do what their peers do, and they like to have fun etc. (18).

Second, the self-medication hypothesis has not been supported epidemiological tests of it. Some epidemiological studies have only included data from participants who did not report psychotic symptoms before they began to use cannabis [e.g., (19)]. Others have recruited participants who did not have a history of psychotic symptoms [e.g., (20)] while other studies have statistically controlled the association for the effects of a prior history of symptoms of mental disorders (21, 22). These studies have generally found that cannabis use more often precedes than follows the onset of psychotic symptoms (9).

Third, in prospective studies, persons with psychoses who used cannabis before their diagnosis, and continue to do so after treatment, have poorer clinical outcomes than those who discontinue cannabis use (e.g., higher rates of relapse and more positive symptoms) (15, 16).

This finding is inconsistent with the self-medication hypothesis.

### Shared risk factors

In epidemiological studies, a history of regular cannabis use in young adulthood predicts an approximate doubling of the risk of developing a psychosis. Skeptics have argued that this size of association could be explained by shared risk factors that have similar sized associations with the risks of using cannabis and of developing a psychosis (7).

The estimated doubling of risk, however, may be attenuated by measurement error. In many studies, for example, cannabis use is simply measured as daily or near daily cannabis use. Epidemiological studies that have used finer grained measures of the type and potency of cannabis suggest that the risk of psychosis is much >2 in persons who use cannabis with high levels of THC and low levels of CBD (10). If the association with cannabis use shows a dose response relationship, then shared risk factors must also show a dose response relationship to both cannabis use and psychosis risk.

Longitudinal epidemiological studies have assessed the shared risk factors hypothesis by controlling and statistically adjusting for plausible confounders, such as, other drug use, personal characteristics that predict psychosis, and a history of psychotic symptoms [e.g., (19, 20, 22–24)]. The number and type of confounding variables has varied between studies. Fixed effects regression has also been used to control for the effects of *unmeasured* confounders (23).

One type of confounding presents challenges for the strategy of statistical control. This is the strong association between cannabis use and tobacco smoking, which is more common among persons who develop schizophrenia than among peers without these disorders (25). The authors of a systematic review of the epidemiological studies of tobacco use in schizophrenia (25) argued that there was good evidence that cigarette smoking plays a contributory causal role in the onset of schizophrenia.

Disentangling the potential causal roles of tobacco and cannabis smoking is difficult because these types of drug use are strongly correlated. Controlling for cigarette smoking may also be inappropriate if tobacco smoking is a contributory cause of cannabis smoking. One analysis of data from the Avon Cohort found that the association between cannabis use and psychosis was greatly attenuated after controlling for cigarette smoking (26). Other studies suggest that tobacco smoking does not explain the association between cannabis use and psychosis [e.g., (27, 28)], including a later follow up of the Avon cohort (28).

Epidemiological studies have also assessed whether the association between cannabis use and psychosis can be explained by shared genetic factors that increase both the risk of using cannabis and the risk of developing a psychotic disorder. A weakness with these genetic studies is that many have only measured cannabis use over the lifetime (or the past year) rather than daily or near daily use over a period of years. These measures limit the statistical power of these studies in testing competing hypotheses. Another weakness of genetic studies is that they have not been able to identify genotypes that accurately predict the risk of using cannabis or developing a psychosis.

Gillespie and Kendler (29) reviewed studies that used a variety of genetically informed research designs to assess genetic contributions to associations between cannabis use and schizophrenia. These included: studies of the size of the association in cohorts of people of varying levels of genetic relationships (e.g., twins, parents, siblings, cousins and unrelated), Mendelian randomization studies, and studies that used polygenic risk scores to adjust the size of the association between cannabis use and psychosis. Gillespie and Kendler argued that these studies have found evidence of shared genetic risks for cannabis use and psychosis. They have also found evidence that emerging symptoms of psychosis increase the risks of using cannabis but concluded, nonetheless, that there is consistent evidence that cannabis use played a small contributory causal role in the development of psychoses.

## Moving beyond policy-biased appraisals of the evidence

Two things are needed to move beyond policy-biased appraisals of the evidence on cannabis and psychosis.

First, we need to use explicit criteria to assess the evidence for contributory causal relationships and apply them in an even-handed and consistent way. We should avoid the example of the tobacco industry in setting such a high standard of evidence for a causal inference that no evidence can satisfy it (30). We should also avoid accepting weaker evidence in support of causal explanations, for example accepting observational evidence that persons with psychosis who use cannabis have better social adjustment than those who do not as evidence of the cognitive benefits of cannabis use [e.g., (31)].

Second, we need more nuanced analyses of the relationships between evidence and policy than those often implicitly assumed [e.g., (32, 33)]. For example, accepting that regular cannabis use may play a contributory causal role in psychosis does not entail support for cannabis prohibition. There is experimental evidence, for example, that heavy alcohol use is a contributory cause of the psychosis delirium tremens (34). There is also observational evidence that sustained heavy alcohol use can produce psychoses that persist beyond alcohol withdrawal (35, 36). This evidence does not justify alcohol prohibition because policy makers have to consider the social and economic consequences of the policy, as revealed during national alcohol prohibition in the USA from 1920 to 1933 (37).

Ideally democratic pluralist societies should decide on an appropriate cannabis policy by weighing the costs and benefits of cannabis use and cannabis control policies (38, 39). Policy makers need to weigh the harms that may arise from cannabis prohibition, such as, criminal records for cannabis users, production of a large illicit market, police corruption and discriminatory enforcement of the criminal law (38). The costs of cannabis prohibition and the potential benefits of regulating and taxing cannabis have led a majority of US citizens to support the legalization of adult cannabis use (40).

If a government decides to legalize cannabis, however, the evidence on cannabis and psychosis is relevant in making decisions as to how cannabis should be regulated. Experience with alcohol (41), for example, suggests that we should discourage the use of high potency cannabis by basing taxes on the THC content of cannabis products or setting a cap on their THC content (42). The availability of cannabis retail outlets could also be limited and restrictions on the legal age of purchase enforced to reduce adolescent access (41, 43).

## Data availability statement

The original contributions presented in the study are included in the article/supplementary material, further inquiries can be directed to the corresponding author.

## Author contributions

The author confirms being the sole contributor of this work and has approved it for publication.
